# Extramedullary Plasmacytoma of the Tonsil

**DOI:** 10.1155/2011/430809

**Published:** 2011-09-22

**Authors:** Kevin C. Huoh, Annemieke Van Zante, David W. Eisele

**Affiliations:** ^1^Department of Otolaryngology-Head and Neck Surgery, University of California, San Francisco, CA 94115, USA; ^2^Department of Pathology, University of California, San Francisco, CA 94143, USA

## Abstract

Plasma cell tumors are a diverse group of neoplasms characterized by monoclonal proliferation of plasma cells. Extramedullary plasmacytoma (EMP) is a rare form of localized plasma cell tumor that arises most often in the head and neck region. We present an unusual case of EMP of the palatine tonsil from a tertiary care university hospital. We discuss the histopathologic and radiologic evaluation as well as treatment of EMP.

## 1. Introduction

Extramedullary plasmacytoma (EMP) is a rare plasma cell neoplasm that occurs outside of the bone marrow [1]. EMP usually occurs in the head and neck region (80%), with the nasopharynx and sinonasal cavities being the most common sites [2, 3]. Involvement of the tonsil is unusual [1, 4]. In this paper, we present a case of solitary tonsillar EMP. 

## 2. Case Report

A 52-year-old man presented with a left tonsil mass. The patient was an amateur vocal performer and had noted tonsillar asymmetry during singing exercises. Except for mild odynophagia, the patient was asymptomatic. His medical history was unremarkable other than longstanding obstructive sleep apnea and obesity. He had a remote history of smoking and occasional alcohol use. Examination revealed an exophytic lesion of the left palatine tonsil. There was no cervical lymphadenopathy.

**Figure 1:**
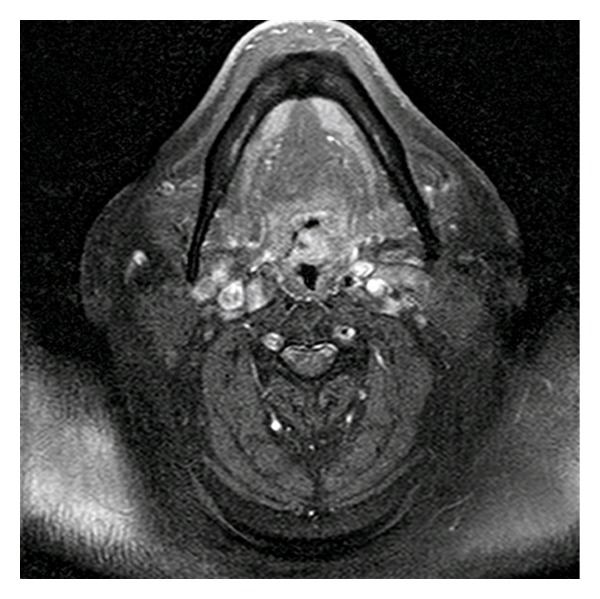
Axial T1 weighted contrast-enhanced MRI image shows left oropharyngeal mass.

Magnetic resonance imaging (MRI) demonstrated a 2.1 cm mass of the palatine tonsil with associated enhancement (Figure 1). Positron emission tomography (PET) was negative for lesions aside from the tonsil. A biopsy of the left tonsil was performed. 

Histologic evaluation showed focal mucosal ulceration, a plasma cell infiltrate, and deposition of amorphous eosinophilic material. A congo red stain was performed, and the amorphous material showed apple-green birefringence characteristic of amyloid. Immunoperoxidase staining showed the plasma cells to be lambda light chain restricted, consistent with a monoclonal process. A diagnosis of extramedullary plasmacytoma with amyloid deposition was made.

**Figure 2:**
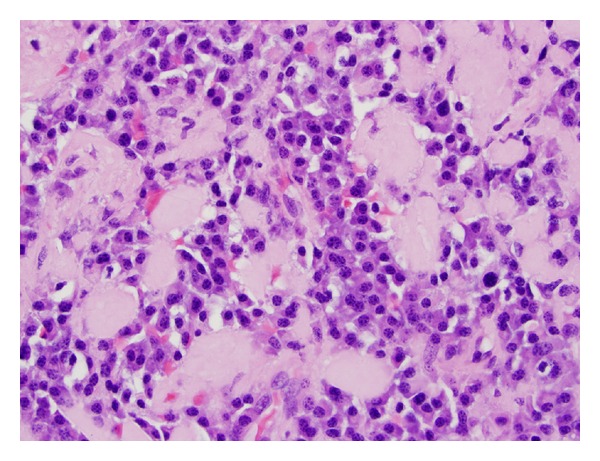
Photomicrograph shows infiltrate of mature plasma cells with associated amorphous eosinophilic amyloid deposits (hematoxylin and eosin stain, 600x magnification).

The patient underwent complete surgical resection of the left tonsil. Final pathology was consistent with EMP (Figure 2). Hematologic evaluation for plasma cell myeloma and systemic amyloidosis was negative. No adjuvant treatment was recommended, and the patient remains clinically free of disease after 6 months of followup.

## 3. Discussion

EMP is a plasma cell neoplasm that occurs predominantly in the upper aerodigestive tract [4]. Most lesions of the head and neck occur in the sinonasal region [1]. Our patient presented with isolated disease of the palatine tonsil which is rare. In a previously reported single-center series of 68 patients with EMP of the head and neck, only 13 cases occurred in the oropharynx [2]. A larger retrospective analysis found 10.5% of 714 cases occurred in the palatine tonsil [5]. 

While inhalant exposure has been proposed as a risk factor for EMP of the head and neck, evidence to support this has been inconclusive [4]. Patients with EMP of the tonsil present with symptoms referable to unilateral tonsil enlargement including obstructed breathing. In our patient, there was a history of obstructive sleep apnea but this was longstanding and likely related to obesity. 

Diagnosis of EMP can be made by tissue biopsy or a fine needle aspiration (FNA) biopsy. Reports have highlighted the challenges in diagnosis by FNA due to similarities with inflammatory conditions [1]; however, FNA biopsy is useful in order to exclude other diagnostic considerations including squamous cell carcinoma. If FNA is used in conjunction with flow cytometry or immunohistochemical analysis, a conclusive diagnosis of EMP can be made [1]. 

Once a diagnosis of EMP is made, further workup includes imaging and hematologic evaluation. MRI is useful in determining the extent of local disease, response to treatment, and in the detection of recurrence [1, 3]. PET scans are useful in the detection of distant sites of disease suggesting systemic plasma cell myeloma. Hematologic evaluation is recommended, and workup generally includes a complete blood count, serum and urine protein electrophoresis, quantitative immunoglobulin determination, bone marrow biopsy, and a skeletal survey [2]. All of these tests were negative in our patient.

Histologic examination of EMP usually shows a monotonous infiltrate composed of discohesive plasma cells characterized by eccentrically placed round nuclei with coarse clumpy chromatin. Immunochemical staining reveals reactivity for either the lambda or kappa immunoglobulin light chain and establishes monoclonality [1, 4, 5]. 

Treatment approaches include surgery and/or radiation therapy [3]. EMPs respond well to radiation therapy and some advocate use of radiation as primary treatment [3, 4]. When disease is localized and amenable to complete resection, surgery is advocated. In our case, the lesion was easily accessible, and complete surgical removal was accomplished. Long-term posttreatment surveillance is recommended as recurrent disease and progression to disseminated plasma cell myeloma can occur [5].

## References

[1] Sakai Y, Ikeya J, Morita I, Sato K (2008). Extramedullary plasmacytoma of the tonsil diagnosed by fine-needle aspiration cytology. *Annals of Diagnostic Pathology*.

[2] Bachar G, Goldstein D, Brown D (2008). Solitary extramedullary plasmacytoma of the head and neck—Long-term outcome analysis of 68 cases. *Head and Neck*.

[3] Miller FR, Lavertu P, Wanamaker JR, Bonafede J, Wood BG (1998). Plasmacytomas of the head and neck. *Otolaryngology—Head and Neck Surgery*.

[4] Tokatli F, Puyan FO, Alas RC, Tuncbilek N, Uzal C (2008). Extramedullary plasmacytoma: clinicopathology, immunohistochemistry and therapeutic approach to a case with a tonsillar site. *Hematology/Oncology and Stem Cell Therapy*.

[5] Alexiou C, Kau RJ, Dietzfelbinger H (1999). Extramedullary plasmacytoma: tumor occurrence and therapeutic concepts. *Cancer*.

